# What Can We Do to Increase the Attendance at Our Dental Societies?

**Published:** 1897-05

**Authors:** S. M. White

**Affiliations:** Benton Harbor, Mich.


### THE DENTAL REGISTER.

VOL. LI.] MAY, 1897. [No. 5.

### COMMUNICATIONS.

### What Can We Do to Increase the Attendance at Our Dental Societies?

BY S. M. WHITE, D.D.S., BENTON HARBOR, MICH.

Read before the Dental Society of Southwestern Michigan, at Kalamazoo, April 13, 1897.

*Mr. President, Officers and Members of the Southwestern Michigan Dental Society:*

"What can we do to increase the attendence at our dental societies?" was proposed by the American Dental Association.

In order that we might have a more thoughtful paper on the subject, I invited ideas and suggestions from a few leading members of our profession. I hope to bring forth a few thoughts for this body to discuss. It seems that the dental societies ought to increase in attendance. We are not all of the same disposition, nor have we the same tastes. When this is duly considered it seems a wise provision, for the saying, "Variety is the spice of life," is also applicable here.

But this may prove a source of much trouble under certain circumstances. The danger is that those of like tastes and similar dispositions will form cliques, which are harmful to any society. Of course, we do not urge that we must make a bosom friend of every member of the society; but in our gatherings there should be a general cordiality—none should have the cold shoulder, and none be treated indifferently. If your stock of intelligence is somewhat greater than that of your fellow, do not make him feel your superiority, and thus repel him; but rather treat him in such a manner that he would enjoy being with you. Teach and instruct him all the while, but do it in such a way as though you were learning from him. Seek to advance each individual's interest, and you will advance the interest of the whole society.

We have in this State over eight hundred dentists that are recognized by the Board of Examiners. Can it be possible that 675 do not need the benefits derived from attending dental societies? Twenty-five thousand dentists in the United States, and only 5 percent giving dental societies any attention. Something is wrong.

Gentlemen, I believe the members of the dental societies will have to do some missionary work if we improve on this line. We should have a dental society in every congressional district, and ought to have local societies in every city of ten thousand or more. Meetings of this kind to elevate our profession ought to be an inspiration to every man. I have never heard any man say, I regret having used time attending this society. Have you? If all the dentists would turn out as well as they do in Southwestern Michigan there would be six or seven hundred present, and when the State society would meet the city would then know the dentists were out. We believe a cordial invitation ought to be sent to every practicing dentist within reach of the place of meeting; that every member of the society ought to be a committee of one to invite personally every dentist in his city, town, or vicinity, to attend; urge and bring them if *possible.*

There should be a committee appointed to introduce the new men that attend our societies to every man present, and not allow them to feel they are not welcome, as is often the case in other societies. The visitors should be invited and expected to speak on subjects discussed, as well as the members. Why not?—they are are our invited guests. Let us be a little more zealous in our cordiality. If they are welcome to enter into the discussions and enjoy all privileges, they surely will feel that we want them with us.

Dentists are a little timid about pushing themselves forward as well as other people. Last June the State Association met at

Grand Rapids. How many in this meeting attended? There are 50 dentists in Grand Rapids, and not over 20 percent were there. One of that number sent his name in to become a member, but was not received. He was a man of good moral character, and has built up a good business. What was the reason? It was alleged he used to advertise. Please show me one man out of the 140 that are in good standing, members of the State Association, that do not advertise in some way, and I will show you 139 that do, in a professional way, of course. Perhaps this dentist had done more than some of the society members; but when he wanted to advance they shut down on him—the wrong kind of a spirit to build up dental associations. Why not recognize all dentists that the State Board recognizes?

We have in this State over eight hundred dentists that the State Board of Dental Examiners recognizes as eligible to practice; there were in attendance at Grand Rapids about 125. What was the reason more in the State did not attend, particularly members of the Association? Some member of the State Association please answer.

A member of the State Association was invited before the Board of Censors and informed that he could not place in his card a cut, "Teeth Without Plate." He kindly informed the committee that he considered the card perfectly legitimate, and that he would withdraw from the Association. They should broaden their views, and not let a few minor points injure the attendance and interest in the State Association.

In answer to my request, John A. Gwynne, D.D.S., Chicago, Ill., writes: "The first thought to be instilled in the minds of our brother dentists is the very important one that money-making is not all that is expected of us in our profession, by any means; it has indeed a higher and nobler aim in this life, and, I assure you, all should have in the first place *deep love for our work*, or all else fails. Each and every one in our profession should feel it his duty, as well as a pleasure, to be able to attend our meetings —should make all *sacrifices possible in order* to attend—instead or hunting up some pretext for remaining away. Generally, I am sorry to say, it is the influence of that almighty dollar or two, as the case may be. In order to draw, each must cultivate the desire to aid this all-important work by doing all in his power to advance such meetings by making them more attractive, more sociable, an occasion of good feeling and good cheer, by extending the hand of good-fellowship with a kindly smile of deepest warmth, *not* that *cold, haughty* stare of superior importance, vainly assumed by far too many of all professions. Then will I promise you a better co-operation of many more on the outside—brethren who now fail to take an active interest with us. And last, but not least, we must make it our personal aim to make each and all at *home*, and see that he has enjoyed himself, as well as being benefitted, by having attended such meeting. And I promise you, gentlemen, he will return again, and, more than likely, bring a friend with him at the next society meeting."

W. C. Y. Ferguson, D.D.S., Paw Paw, Michigan, says: "In the first place, 'the code of ethics' is not liberal enough in many cases. Again, a lack of *friendliness* and *courtesy* is shown by the leading members of the Association to those who are comparatively strangers. Also, many think they can spend their time more profitably (get more pointers) at the office of some expert, or at a post-graduate college, especially when they see many who claim to attend our State Association present at very few of its sessions, using the time as an outing, if I may so term it, but have it announced in their local papers of course, that they are attending the Dental Association, etc. Professional duties often prevent regular attendance, so does the matter of cash, also location of place of meeting."

J. C. Walton, D.D.S., Howell, Michigan, replies:

"All want to known how to diminish failures, to promote co-operation, to facilitate transmission of ideas to our clientele, to secure proper status, to guard against financial loss; such would be general progress.

"We may have the benefit of contact between man and man—which is always a powerful attraction—but it should only be an effort for research, experiment and teaching. It is right to compete with and combat quackery, but wrong to ignore and shun the quack. The way to correct newspaper deception is to supply newspaper education. Quackery should be regarded as misdirected energy, and a thoughtful effort made to get control of it. If we are not willing to tolerate it, to listen to it, to find out the truth that may be in it, to supplement it, to enlighten it, we belong back in the long-gone period of history. We may be sure there is some reason for its existance. Why ignore a reason wherever found? The savage remains the savage, the thoughts of the savage continually being within him. Place him in the midst of civilization, give him new thoughts, new hopes, new aspirations, new impulses, and you raise him above the savage state. This is the whole history of civilization, of enlightenment. Three-fourths of us need to be placed in the midst of professional civilization; we need to be given new thoughts, new hopes, new aspirations, new impulses and raised above ourselves. Ignorance and enmity are convertible terms. Rancor and retaliation are likely to make trouble until transformed by concession and education. Increased knowledge has never brought aught but blessing to the world. Membership in the society would strengthen any dentist's attachment to his profession. In every State are needed one or more societies that are founded on those broad principles which find their counterpart in the heart of every dentist: a society instituted and managed for no personal or selfish gain; a great school of comparative dentistry, to bring different methods and men in contact and conference, and to encourage and deepen the spirit of brotherhood; to emphasize the distinctive achievements of the art; to bridge the chasm of ethical separation between the liberal and the exclusive elements, and induce dentists to pull together for our common improvement.

"There is little use of cultivating sentiment against the dentist of the people, refusing to educate the masses, and then expecting him to fraternize on present society terms. It is the business of societies to teach and demonstrate the beauties of an ethical life, the profit of correct practice, and the nobility of our calling. If a college education and society affiliations do not develop courtesy for the opinions of others, toleration, a spirit of helpfulness, and an earnest desire to do one's best, they fail of a high mission.

"Electricity sleeps in mere potentiality, as a force hid in the coal bin, until, through heat and motion under proper control, it appears to work its wonders in your office; so, the genius of human power lies dormant until shocked into efficiency by a wave of suggestion or a flash of illumination. At a dental meeting a single word, the turn of a phrase, a demonstration, may be potent to stir into activity a native dormant power. Who would deny any member of our profession the inspiration? Every dentist should experience the thrill of such an inspiration. Who of you have attended a meeting without having had the deepest wells of your thought strangely moved to action, and, going home with energy awakened in every fiber, have not felt irresistibly the impulse to do better? Yes, the present age of enlightenment demands this reciprocal action, this interchange of intellectual currents between dentist and dentist. Wisdom so inspired has given to the world all that is worthy in art, science, letters and all else that is imperishable in civilization."

C. R. Rowley, D.D.S., Chicago, Illinois, says:

"How best to increase the attendance at Dental Societies has been a question to dentists ever since such a thing as a dental society was organized.

"First. To make them attractive by plenty of good papers and pleasure in the way of banquets, rides about the city in which the meetings are held.

"Second. Embrace all the territory possible, the Southwestern Michigan Dental Society should include Northern Indiana and Chicago.

"Third. Take special pains to invite dentists not in the habit of attending dental meetings, and give them special attention when they do attend, make them feel at home and their presence adjunct to the society. Many dentists labor under the impression that they are not wanted at dental societies; they are not recognized by the officers and seldom attend a meeting more than once —when a little cordiality would make devoted attendants.

"Fourth. I would have interpersed between the readings and discussions of each paper, a call for 'incidents of office practice' as no paper can be written and subject discussed pertaining to dentistry without incident of office practice being brought out by many in attendance.

"Fifth. I think the S. W. D. S. of Michigan is to be complimented; but few societies ever started with a more promising future, and the attendance so far has always been good."

M. A. Mason, D.D.S., Fort Wayne, Indiana, says:

"What can we do to increase the attendance at our Dental Societies?

"In reply to your question of some days ago I trust you will permit me to be brief.

"Only those who attend dental societies fully appreciate their value to themselves, their profession and their patients. If we can demonstrate to some of our professional brethren who do not attend societies that we do learn things of value to us in our practice, that we do serve our patrons better, that they appreciate more highly our services for our endeavor to keep abreast of the times, we will be able to reach some very desirable members. A few others can be reached by persistently inviting them until they decide to go to see what is done at the society meetings.

"That this work may be done systematically and thoroughly, it would not be a bad idea for a number of the members to agree among themselves to add a certain number to the society at the coming session. Then if each member of this self-created committee did its work well I believe that a large membership could be added each session.

"We have a plan something like the above at work in the Isaac Knapp Dental Coterie. A few, who can nearly always be relied on to attend, have constituted themselves a committee to look after those who are sometimes forgetful of the Coterie's interest and welfare. The result is that our society which has a membership of eleven has the splendid general average of about eight.

"But it is not all to have a dentist put in his appearance at a dental society. If you would retain him long give him something to do. Let him feel that he has in some way added to the interest of the meeting, that he has said something or done something for the society. Some men can write, some can give clinics, some can talk, while some would be better satisfied to work for the society in an official way. Aim by all means to have as few wall flowers as possible and the greatest number will go home with the feeling that the society was all the more interesting for the part they took in the program.

"The program should be arranged to please all if possible. Papers, discussions and clinics are all indispensable, and yet these should be arranged to please the greatest number.

"Summarizing, I would say: Demonstrate to your patients and fellow-practitioners that the society is of value to you in your practice. This constitutes an unanswerable argument in favor of the society.

"Then systematically and thoroughly talk society. Those dentists you can't talk to, write to. Advertise, advertise the society—once, twice, a dozen times, if necessary. Don't let any one have the excuse of not knowing when and where the meeting is to be. Have a program worth listening to and have *all* take part in its execution. Distribute the offices as freely as possible."

Gustavus North, A.M., D.D.S., Springville, Iowa, writes:

"To increase the attendance of our dental societies is one of the difficult problems with which our profession has to deal. I believe it is the desire of all dentists to do right and to be respected by his professional brethen.

"Dental societies are often times organized with a selfish motive by men who are working for popularity, and think '*big I and little u*,' such men are very objectionable to some members of the profession, and have a tendency to scatter more than gather. *Love* has power. 'Love thy neighbor as thy self.' Love will give strength and accomplish great good.

"Give all dentists a welcome invitation to attend societies, sometimes a personal letter of welcome will move some timid one, and encourage him to attend. Let your meetings be a social as well as an educational gathering, always make a special effort to cultivate a brotherly and welcome feeling with every visiting member. Men are sensitive beings and always appreciate kindness and respect.

"I have attended dental societies where I have been made very welcome—such a free, brotherly love that all worked in harmony and I felt honored to be in attendance. And again I have attended societies where such a cold, distant feeling existed that it was unpleasant.

"I believe the attendance of all societies can be increased if men will lay all selfishness aside and endeavor to cultivate a true loving spirit. Oh, how men appreciate love and kindness! I try to love and respect all, let the circumstances be what they may.

"So, in conclusion, let your motto be 'Love.' Cultivate true friendship, love one another and your society will be crowded with members."

